# Supplementary figures and images for: Choline regulation of triglycerides synthesis through ubiquintination pathway in MAC-T cells
Source: PeerJ. 2023 Dec 19;11:e16611. doi: 10.7717/peerj.16611 (PMC10740596; doi:10.7717/peerj.16611)

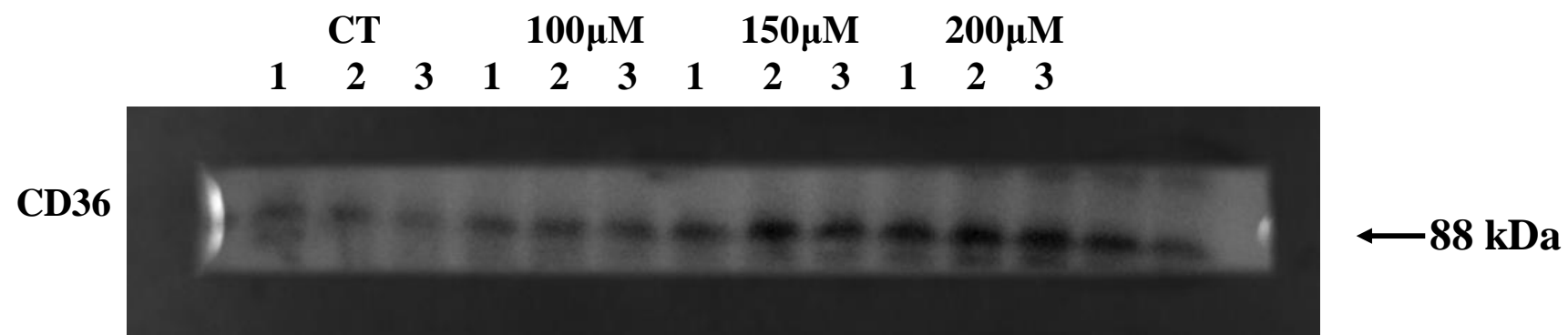

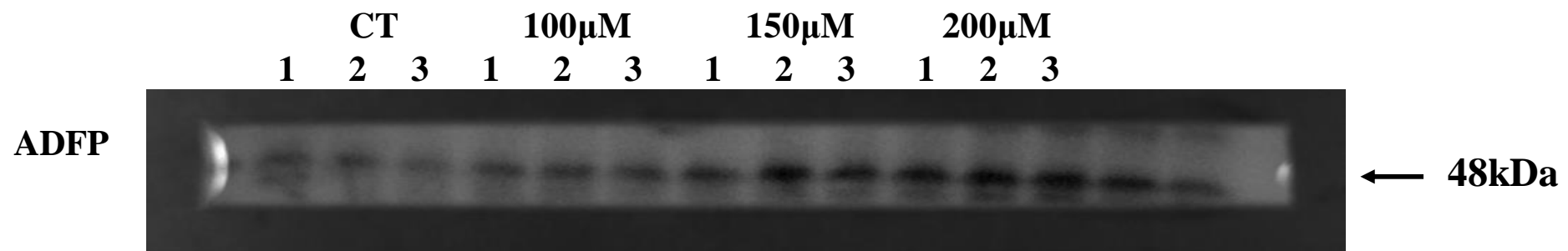

| CT |   |   | 100μM |   |   | 150μM |   |   | 200μM |   |   |
|----|---|---|-------|---|---|-------|---|---|-------|---|---|
| 1  | 2 | 3 | 1     | 2 | 3 | 1     | 2 | 3 | 1     | 2 | 3 |

UB

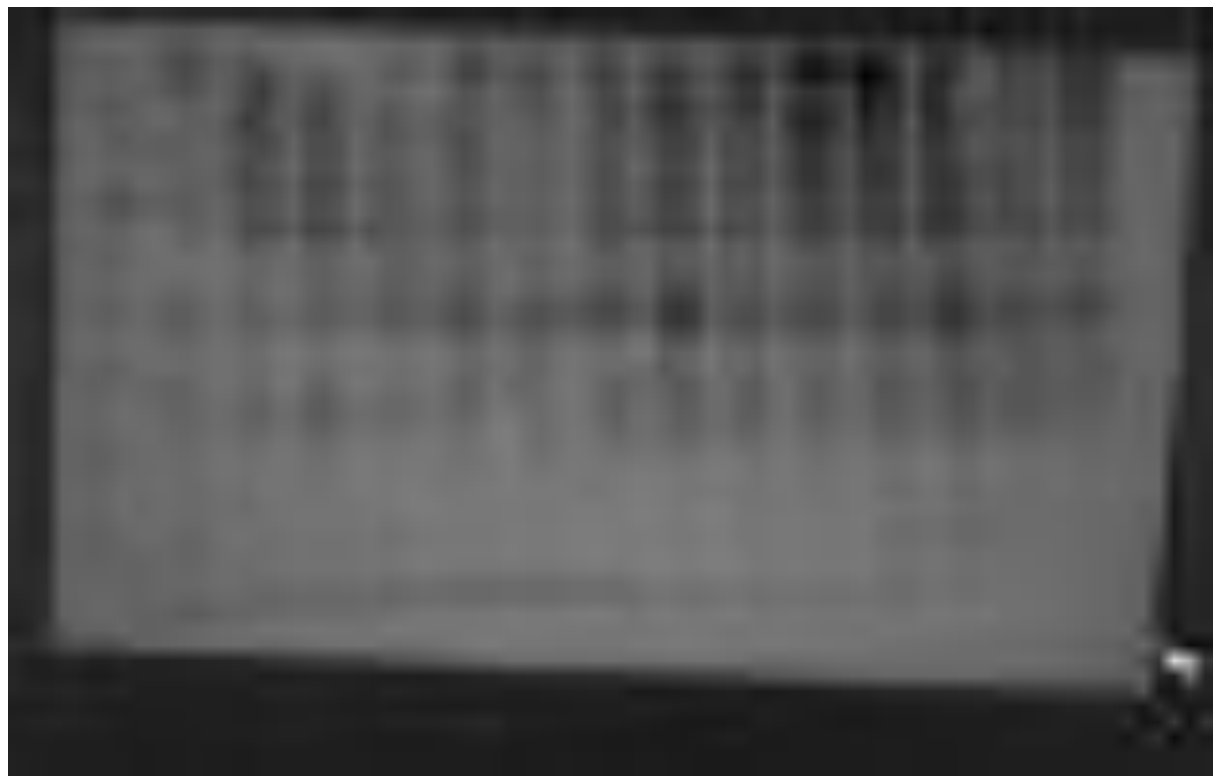

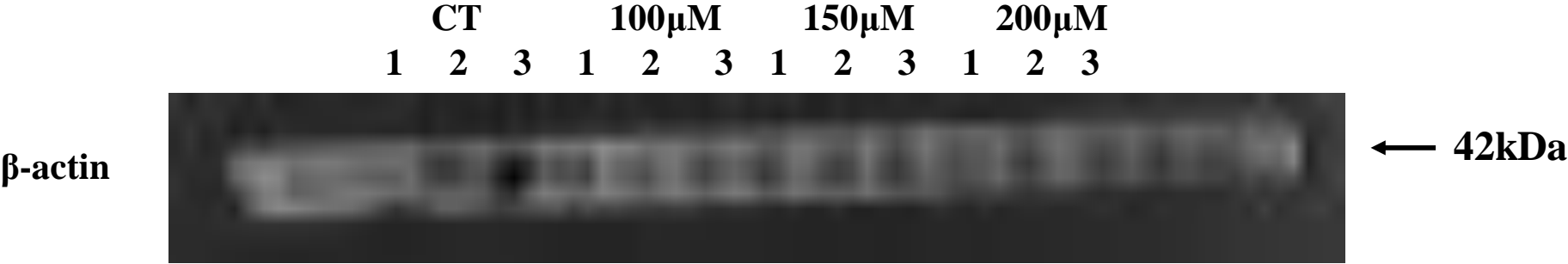

Supplement: Supplemental Information 1 [file peerj-11-16611-s001.pdf]
